# The design of a randomized, placebo-controlled, dose-ranging trial to investigate the efficacy and safety of the ADAMTS-5 inhibitor S201086/GLPG1972 in knee osteoarthritis

**DOI:** 10.1016/j.ocarto.2021.100209

**Published:** 2021-08-16

**Authors:** Olivier Imbert, Henri Deckx, Katy Bernard, Ellen van der Aar, Maria Pueyo, Nadeem Saeed, Thomas Fuerst, Wolfgang Wirth, Philip G. Conaghan, Felix Eckstein

**Affiliations:** a Institut de Recherches Internationales Servier (IRIS), Suresnes, France; b Galapagos NV, Mechelen, Belgium; c Bioclinica, London, UK; d Bioclinica, Newark, CA, USA; e Chondrometrics GmbH, Ainring, Germany; f Institute of Anatomy and Cell Biology and Ludwig Boltzmann Institute for Arthritis and Rehabilitation (LBIAR), Paracelsus Medical University, Salzburg, Austria; g Leeds Institute of Rheumatic and Musculoskeletal Medicine, University of Leeds and NIHR Leeds Biomedical Research Centre, UK

**Keywords:** Knee osteoarthritis, Cartilage, Clinical trial, Disease modification, Quantitative magnetic resonance imaging, ADAMTS-5 inhibitor

## Abstract

**Objective:**

This study aims to assess the efficacy of the anticatabolic ‘a disintegrin and metalloproteinase with thrombospondin motif-5’ (ADAMTS-5) inhibitor, S201086/GLPG1972, in slowing cartilage loss in participants with knee osteoarthritis (OA).

**Design:**

ROCCELLA (NCT03595618) is a randomized, double-blind, placebo-controlled, parallel-group, dose-ranging, phase 2 trial. We plan to enrol a total of 852 participants with knee OA across 12 countries. Participants will be randomized 1:1:1:1 to receive 75, 150 or 300 ​mg S201086/GLPG1972, or placebo orally, once daily for 52 weeks. Eligible participants will be aged 40–75 years and have predominantly medial knee OA with centrally read Kellgren–Lawrence grade 2 or 3, OARSI atlas medial femorotibial joint space narrowing grade 1 or 2, and consistent moderate to severe baseline pain. The primary endpoint will be the change from baseline to week 52 in magnetic resonance imaging-assessed central medial femorotibial compartment cartilage thickness. Secondary endpoints will include other structural outcomes, and patient-reported outcomes, as well as safety and pharmacokinetic assessments. Study sites will be assessed for eligibility based on factors including imaging quality, and images will be centrally read and quality checked.

**Conclusions:**

Using strict inclusion criteria and leading imaging techniques with stringent quality controls, the ROCCELLA trial will evaluate the efficacy of S201086/GLPG1972 in slowing cartilage loss in participants with knee OA. The selected eligibility criteria should enrich for participants with OA who experience sufficient cartilage loss to allow detection of a substantial treatment effect.

## 1 Introduction

The degradation of cartilage extracellular matrix components, primarily aggrecan and collagen, leading to cartilage loss is a central feature of osteoarthritis (OA). Preclinical studies have demonstrated that cleavage of aggrecan by the aggrecanase ‘a disintegrin and metalloproteinase with thrombospondin motif-5’ (ADAMTS-5) is important in the pathogenesis of OA [1,2]. Deletion of the ADAMTS-5 catalytic domain reduces cartilage degradation in a mouse model of OA [3], and *Adamts5* null mice appear resistant to OA-associated pain [4]. Concentrations of alanine-arginine-glycine-serine (ARGS) fragments, products of aggrecan cleavage by ADAMTS-5, are increased in the synovial fluid of patients with knee OA and following acute knee injury [5].

Several pharmaceutical agents have been investigated as disease-modifying osteoarthritis drugs (DMOADs) for knee OA [[6], [7], [8], [9], [10]]; however, to date, none have received regulatory approval. S201086/GLPG1972 is a potent and highly selective inhibitor of ADAMTS-5 in development as an orally administered DMOAD. In *in* *vitro* pharmacological studies, S201086/GLPG1972 selectively inhibited ADAMTS-5 and triggered reduced concentrations of anticatabolic biomarkers in mouse and human explants [[11], [12]]. Furthermore, in rodents with surgery-induced OA, oral administration of S201086/GLPG1972 post surgery resulted in significant DMOAD activity, as measured by cartilage structural damage scores, proteoglycan content, fibrillation index, subchondral bone sclerosis and osteophyte size [[11], [12]].

Following the success of these investigations, a first-in-human study was performed to assess the safety, pharmacokinetics and pharmacodynamics of S201086/GLPG1972 in healthy male participants [13,14]. Treatment was generally well tolerated, after a single oral dose (up to 2100 ​mg) and daily oral doses (up to 1050 ​mg) for 14 days. S201086/GLPG1972 was rapidly absorbed and eliminated, with an overall mean apparent half-life of 10 ​h. In addition, plasma concentrations of N-terminal ARGS neoepitope fragments decreased progressively over 14 days of S201086/GLPG1972 treatment, by up to 60% from baseline; conversely, reductions in serum or plasma ARGS fragment concentrations were not observed in participants treated with placebo [13,14]. Similar results were observed in patients with knee or hip OA exposed to S201086/GLPG1972 100, 200 or 300 ​mg orally once daily for 29 days, confirming that the drug reduced the aggrecanase activity of ADAMTS-5 [13].

Subsequently, the phase 2 ROCCELLA trial was designed to assess the disease-modifying efficacy and safety of S201086/GLPG1972 in the treatment of radiographic, painful knee OA. The primary objective of the study is to demonstrate the efficacy of at least one dose of S201086/GLPG1972 in slowing magnetic resonance imaging (MRI)-assessed cartilage loss in the central medial femorotibial compartment (cMFTC) compared with placebo over 52 weeks in participants with knee OA.

## 2 Materials and methods

### 2.1 Study design and setting

**Fig. 1:**
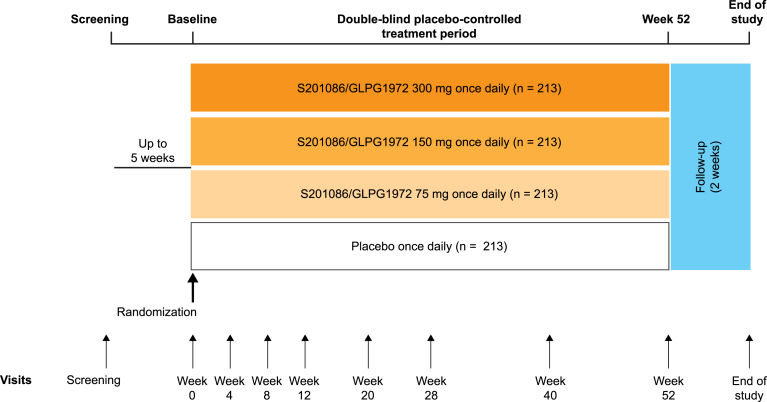
Study design.

ROCCELLA is a randomized, double-blind, placebo-controlled, parallel-group, dose-ranging phase 2 study (NCT03595618) that aims to recruit 852 participants across 12 countries: Argentina, Brazil, Canada, Denmark, Hungary, Japan, Poland, Russia, South Korea, Spain, Taiwan and the USA. The study design is shown in Fig. 1. The study will comprise a screening period (up to 5 weeks from the screening visit to baseline), a double-blind treatment period (52 weeks) and a follow-up period for safety (with an end-of-study visit 2 weeks after treatment completion or early study discontinuation). Participants will be randomly assigned 1:1:1:1 to 75, 150 or 300 ​mg S201086/GLPG1972 or placebo orally, once daily. The final protocol and its amendments will be reviewed and approved by the institutional review board or independent ethics committee at each participating centre. The study will be conducted in accordance with the Declaration of Helsinki. All participants must provide written informed consent.

Block randomization will be via an Interactive Web Response System (IWRS) and stratified by geographical zone (Japan, South Korea/Taiwan and rest of the world). A centralized decoding system within the IWRS will be used for unblinding in the case of an imperative, justified medical reason.

A schedule of study procedures is shown in Table 1. Treatment will be discontinued at week 52 and participants will attend an end-of-study visit 2 weeks after their last treatment dose. Participants who withdraw will attend a visit at study discontinuation and end of study 2 weeks later, unless they withdraw consent. Reasons for participant withdrawal are listed in Table 2.

**Table 1:** Schedule of study procedures.

Procedure	Screening	Baseline	Week							Premature withdrawal	Study end
			4	8	12	20	28	40	52		
MRI^a^		X					X		X	X^b^	
X-ray^c^	X								X	X^d^	
WOMAC questionnaire		X			X		X	X	X	X	
Pain intensity^c^ (VAS)	X	X	X	X	X	X	X	X	X	X	X
PGA (VAS)		X			X		X	X	X	X	
Analgesic consumption		X	X	X	X	X	X	X	X	X	X
Adverse events	X	X	X	X	X	X	X	X	X	X	X
Vital signs	X	X	X	X	X	X	X	X	X	X	X
Laboratory values	X	X	X	X	X	X	X	X	X	X	X
Physical examinations	X	X	X	X	X	X	X	X	X	X	X
Body weight	X	X			X		X		X	X	X
12-lead ECG	X	X	X				X		X	X	X
Blood samples for PK analysis			X^e^		X^e^		X^f^	X^g^	X^e^	X^e^	

ECG, electrocardiogram; MRI, magnetic resonance imaging; PGA, patient global assessment; PK, pharmacokinetic; VAS, visual analogue scale; WOMAC, Western Ontario and McMaster Universities Osteoarthritis Index.

a Evaluated for the target knee only. The assessment at baseline was performed after obtaining results of screening criteria and before the first study treatment intake.

b To be performed only if the previous MRI (at baseline or week 28) is carried out ≥2 months before the premature withdrawal visit.

c Evaluated for both knees at screening and for the target knee at all other time points.

d To be performed only if the previous X-ray (at screening) is carried out >9 months before the premature withdrawal visit.

e Pre-dose sample (at week 52 and premature withdrawal, no dose is taken).

f Pre-dose sample and one post-dose sample (2–4 ​h post dose).

g Post-dose sample (4–8 ​h post dose). Participants had to take study treatment at least 4 ​h before sampling and report the time of dosing to the study staff.

**Table 2:** Reasons for participant withdrawal.

Reasons for withdrawal
Study treatment must be discontinued by the investigator and the participant must be withdrawn from the clinical study (preferably after discussion with the monitor, who may consult and must inform the sponsor's study physician if applicable) for any of the following conditions: • Life-threatening AE or a SAE that places the participant at immediate risk • Confirmed pregnancy • The following ECG and/or laboratory parameter abnormalities: o QTcF >500 ​ms or a delta >60 ​ms over baseline value (inclusion) on at least two separate ECGs o Increase in liver function tests: 1. ALT or AST >8× ULN (discontinue the treatment immediately) 2. ALT or AST >5× ULN for more than 2 weeks (a decision to stop the study drug should be taken after a confirmed retest) 3. ALT or AST >3× ULN and (total bilirubin >2× ULN or INR >1.5) 4. ALT or AST >3× ULN with the appearance of fatigue, nausea, vomiting, right upper quadrant pain or tenderness, fever, rash and/or eosinophilia (>5%) 5. Presence of evocative clinical symptoms such as jaundice o In general, an increase of serum aminotransferase to >3× ULN should be followed by repeat testing, preferably within 48 h of the laboratory results being received by the investigator of all four of the usual serum measures (ALT, AST, ALP and TBL) to confirm the abnormalities. Based on the re-test results, it should be determined together with monitor if the discontinuation criteria are confirmed. If confirmed, the participant must be withdrawn from the clinical study. In any case of liver toxicity, additional investigations are needed (such as: assessment of alcohol or recreational drug intake, hepatitis infection, etc.) • Any AE or any condition incompatible with continuation of the IMP according to the judgement of the investigator The investigator may also decide to stop treatment with the IMP (preferably after consultation with the sponsor's study physician if applicable) for any of the following reasons: • Use of concurrent therapy that was not permitted • Noncompliance with the IMP treatment, including overdose • Noncompliance with the clinical study procedures • Serious or severe AEs • Worsening of disease condition that in the investigator's opinion needs an alternative treatment approach that is not covered in the clinical study

AE, adverse event; ALP, alkaline phosphatase; ALT, alanine aminotransferase; AST, aspartate aminotransferase; ECG, electrocardiogram; IMP, investigational medicinal product; INR, international normalized ratio; SAE, serious adverse event; QTcF, corrected QT interval; TBL, total bilirubin; ULN, upper limit of normal.

### 2.2 Participant selection

Despite increased understanding of OA, previous DMOAD trials have been unsuccessful. This may have been partly owing to patient populations showing less structural progression over time than expected, thus having insufficient cartilage loss for detection of an anticatabolic treatment effect [15]. In the present study, stringent inclusion criteria will be used to select for participants with knee OA who are expected to experience sufficient cartilage loss in the target knee over 1 year to demonstrate a potential structural benefit of S201086/GLPG1972.

Eligible participants will be aged 40–75 years with a body weight over 40 ​kg and a body mass index of under 40 ​kg/m^2^. Men, and women of non-childbearing potential will be eligible. Participants must have a diagnosis of knee OA based on clinical and radiological American College of Rheumatology criteria (participants must have knee pain, exhibit osteophytes and fulfil at least one of the following: be aged over 50 years, have morning stiffness lasting less than 30 ​min or have crepitus on active motion). Additionally, participants must have had knee pain for at least 6 months and on most days during the month before screening, and have a documented need for symptomatic treatment of the target knee with systemic non-steroidal anti-inflammatory drugs (NSAIDs) and/or other analgesics. To ensure a consistent baseline pain measurement, the target knee must exhibit pain with severity between 40 ​mm and 90 ​mm on a 100 ​mm visual analogue scale (VAS) at both screening and baseline.

**Fig. 2:**
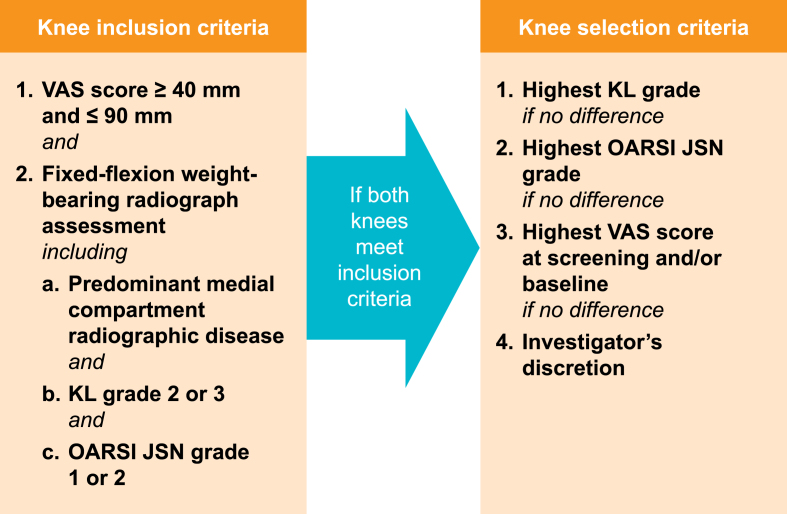
Selection of the target knee. OARSI JSN, joint space narrowing-Osteoarthritis Research Society International; KL, Kellgren–Lawrence; VAS, visual analogue scale.

Target knees must display predominantly medial disease, and have a central readout of Kellgren–Lawrence (KL) grade 2 or 3 radiographic severity and OARSI atlas medial femorotibial joint space narrowing (JSN) grade 1 or 2 [16]. The following features will be assessed for determining predominance of medial compartment disease: relative severity of OARSI JSN (OARSI medial JSN must be greater than OARSI lateral JSN); knee alignment (varus deformity to neutral); and presence of osteophytes and sclerosis. In cases in which both knees fulfil these criteria, an algorithm will be used to select the most affected knee as the target knee (Fig. 2).

Participants will be excluded if they have severe clinical knee malalignment or a knee prosthesis implanted during the year before the start of the study. Participants will also be excluded if they have a medical history of or currently have pathologies other than osteoarthritis affecting the target knee (e.g. septic arthritis, inflammatory joint disease, gout, acromegaly, haemochromatosis, Wilson's disease). Additional inclusion and exclusion criteria are given in Table 3.

**Table 3:** Study eligibility criteria.

Inclusion criteria
1. Male participants or female participants of non-childbearing potential and not breastfeeding. Note: Female participants will be considered to be of non-childbearing potential if they are either surgically sterile (e.g. tubal ligation, hysterectomy) or postmenopausal (at least 12 consecutive months of amenorrhea in the absence of other biological or physiological causes AND 50 years of age or older). 2. Age between 40 and 75 years (both inclusive). 3. Body weight >40 ​kg. 4. Body mass index <40 ​kg/m^2^. 5. Diagnosed for knee OA based on the clinical and radiological criteria of the ACR (documented diagnosis), i.e.: a. Knee pain b. And, at least one of the following: • age more than 50 years • morning stiffness <30 ​min duration • crepitus on active motion c. And presence of osteophytes 6. History of knee pain for at least 6 months and on the majority of days (>50%) during the preceding month. 7. Symptom severity defined by a pain ≥40 ​mm and ≤90 ​mm on a 100 ​mm VAS at screening and inclusion visits (at screening both knees should be assessed for pain and at least one knee should fulfil pain severity defined on this criterion). A knee not meeting the pain criteria at screening must not be eligible as a target knee at inclusion. 8. Documented need for symptomatic as-needed treatment for OA in the target knee with systemic non-steroidal anti-inflammatory drugs and/or other analgesics 9. Disease stage based on a fixed-flexion, weight-bearing X-ray of the target knee∗ and central readout of: a. Predominant medial compartment radiographic disease b. KL grade 2 or 3 c. And OARSI medial femorotibial JSN grade 1 or 2 10. Informed consent
Exclusion criteria
1. Unlikely to cooperate in the study. 2. Participation in another interventional study within 3 months before screening; participation in non-interventional registries or epidemiological studies is allowed. 3. Re-screened participant. 4. Participant unable to understand the study. 5. Poor compliance anticipated by the investigator. 6. Investigator or other study staff or related thereof who is directly involved in the conduct of the study. 7. Severe clinical knee malalignment according to the investigator. 8. Knee prosthesis already implanted (<1 year) or not well tolerated (contralateral side). 9. Knee prosthesis already foreseen within the study period (whichever side). 10. Hip prosthesis recently implanted (<1 year) or foreseen within the study period (whichever side). 11. Previous osteotomy on the inferior limbs (whichever side) other than intervention for hallux valgus with full clinical recovery after surgery. 12. Any surgical operations on the target knee (arthroscopic and non-arthroscopic), other than diagnostic arthroscopy, within the 12 months prior to the screening visit or planned during the study period. 13. Diagnostic arthroscopy of the target knee within the 6 months prior to the screening visit or planned during the study period. 14. Other current or medical history of pathologies affecting the target knee, such as: septic arthritis, inflammatory joint disease, gout, major chondrocalcinosis (pseudogout), Paget's disease of the bone, ochronosis, acromegaly, haemochromatosis, Wilson's disease, rheumatic symptoms due to malignancies, primary osteochondromatosis, osteonecrosis, osteochondritis dissecans, documented severe intra-articular knee injury (e.g. intra-articular fracture), haemophilia, etc. Note: participants with common risk factors for knee OA – e.g. obesity, meniscectomy – are not excluded. 15. Participants with widespread chronic musculoskeletal pain of unclear aetiology (i.e. functional somatic syndromes such as fibromyalgia, with or without previously documented diagnosis). 16. Chronic oral corticosteroid therapy within 1 month prior to enrolment into the study other than stable doses of ≤7.5 ​mg daily prednisolone or equivalent. 17. Corticosteroid or hyaluronic acid intra-articular injections in the target knee in the previous 3 months. 18. Chronic use of medications with and for MMP-inhibitory properties (i.e. tetracycline or structurally related compounds) during the 3 months prior to the screening visit. 19. Bisphosphonates, denosumab, teriparatide, strontium ranelate, romosozumab use (in oral or injectable form) in the previous 12 months. 20. Use of other unapproved drugs for osteoarthritis treatment during the 3 months prior to the screening visit. 21. Chronic use of strong opioids and use of possible drug/drug interaction treatment (CYP3A4, BCRP and 2C19) during 7 days prior to the screening visit. 22. Any contraindication to MRI according to local MRI guidelines, or the inability to undergo a knee MRI exam because of inability to fit in the scanner or knee coil. The presence of a pacemaker or any other implanted electronic devices is considered a contraindication to MRI in this study (accommodations will not be made). 23. Non-pharmacological standard of care for the target knee (physiotherapy, electrotherapy, etc.) if initiated less than 4 weeks before screening visit. 24. Severe or unstable disease of any type that could interfere with safety and efficacy assessments (e.g. uncontrolled cardiovascular, pulmonary, infectious, severe immune deficiency, autoimmune, renal, hepatic, gastrointestinal, endocrine, blood disorders) according to investigator's judgement. 25. History of malignancy in the past 5 years, with the exception of: basal cell carcinoma, resected cutaneous squamous cell carcinoma in situ, prostate cancer in situ with a normal prostate-specific antigen level post treatment, cervical carcinoma in situ, gastric cancer in situ, colon cancer in situ adequately treated with no significant progression over the past 2 years. 26. Class III or Class IV heart failure according to the New York Heart Association classification. 27. Moderate to severe renal impairment; i.e. estimated glomerular filtration rate <45 ​mL/min/1.73 ​m^2^ (Modification of the Diet in Renal Disease formula). 28. The following abnormalities detected on a 12-lead ECG performed at screening visit of either rhythm or conduction (QTcF >450 ​ms for males and >470 ​ms for females, bradycardia with HR ​<50 bpm, measured and stable PR-interval >280 ​ms, or second (except Mobitz type 1) or third degree atrio-ventricular block and complete left branch block) as assessed by central reading. Any other abnormalities are left to the investigator's judgement for final decision. 29. Known severe hepatic impairment (i.e. cirrhosis, active liver disease) or known liver enzymes abnormalities such as: a. Aspartate aminotransferase and/or alanine aminotransferase values ​>2 × ULN b. Alkaline phosphatase >3 × ULN c. Total bilirubin >1.5 × ULN (except in case of Gilbert syndrome). 30. Positive for anti-HIV antibodies, hepatitis B surface antigen or anti-HCV antibodies with a positive test for HCV viral RNA or completion of antiviral HCV treatment ≤12 weeks before screening. 31. Severe malabsorption according to investigator's judgement. 32. Unexplained significant weight loss (>10% of body weight within the last year). 33. Alcohol abuse or drug abuse or addiction according to investigator's judgement. 34. Documented hypersensitivity to the active substance or to any of the excipients (e.g. lactose).
Contraception
Within the frame of this study, as the effect of S201086/GLPG1972 on sperm in man is unknown, male clinical study participants and their female partners of child-bearing potential must use highly effective contraception (as described in the informed consent form) in combination with a barrier contraceptive to prevent pregnancy and to avoid the risk of exposure of the embryo or fetus during the study and up to 12 weeks after the last dose is received. Male participants agree to not donate sperm from the time of first study drug intake during the study until 12 weeks after the last study drug intake.

CYP2C19, cytochrome P450 2C19; ACR, American College of Rheumatology; BCRP, breast cancer resistance protein; CYP3A4, cytochrome P450 3A4; ECG, electrocardiogram; HCV, hepatitis C virus; HIV, human immunodeficiency virus; HR, heart rate; JSN, joint space narrowing; KL, Kellgren–Lawrence; MMP, matrix metalloproteinase; MRI, magnetic resonance imaging; OA, osteoarthritis; OARSI, Osteoarthritis Research Society International; QTcF, corrected QT interval; ULN, upper limit of normal; VAS, visual analogue scale.

∗The target knee (right or left) to be followed up throughout the study: o If both knees fulfil the clinical screening criteria and radiological inclusion criteria, the knee to be chosen should be the most severely affected knee on X-ray (higher KL score); in cases of similar KL scores, the higher JSN score will be selected. o If both knees display the same radiological scores, the knee to be chosen should be the most clinically painful one (higher VAS score at screening). o If both knees display the same radiological scores and are equally painful, the choice should be left to the investigator's discretion.

### 2.3 Interventions

The choice of S201086/GLPG1972 doses was based on pharmacokinetic and pharmacodynamic results in humans, as well as toxicological and pharmacological studies. Specifically, a maximum dose of 300 ​mg was chosen, as daily oral administration of S201086/GLPG1972 300 ​mg or 1050 ​mg in healthy men for 14 days resulted in reductions in ARGS levels versus baseline of 53.4% and 61.4%, respectively, inferring that the 300 ​mg dose resulted in a near maximum reduction in ARGS fragments [13]. The lowest dose was chosen based on findings that the exposure that triggered DMOAD activity in the rat meniscectomy model was a clinical dose of 45 ​mg [12], and in healthy human participants, a single dose of 150 ​mg maintained the plasma concentration above the IC_50_ derived from the human explant assay for almost 24 ​h [13,14].

Both the study drug and placebo will be provided as identical film-coated tablets composed of lactose monohydrate. Participants will take four tablets daily, with one, two or four of these tablets containing S201086/GLPG1972 75 ​mg.

Use of NSAIDs and other analgesics (e.g. paracetamol and tramadol) will be permitted during the study. Other permitted concomitant therapies will include: symptomatic oral drugs (e.g. glucosamine and chondroitin sulphate), provided the dose is stable for at least 12 weeks before baseline and the week 52 visit; intra-articular injection of corticosteroids into the non-target knee if administered more than 4 weeks before baseline and the week 52 visit; and non-pharmacological standard of care in the target knee (e.g. physiotherapy or electrotherapy) if initiated more than 4 weeks before baseline and the week 52 visit. Complete details of permitted and non-permitted concomitant therapies are provided in Table 3.

### 2.4 Outcomes

The primary efficacy endpoint will be the change from baseline to week 52 in cMFTC cartilage thickness of the target knee. Secondary endpoints, including structural efficacy, clinical efficacy and safety endpoints, and exploratory endpoints are listed in Table 4.

**Table 4:** Study endpoints.

Primary endpoint	

	The change from baseline to week 52 in cMFTC cartilage thickness of the target knee (MRI)

Secondary endpoints	

Structural efficacy endpoints	The proportion of participants who have at least 8% cartilage loss in the cMFTC (structural progressors) between baseline and week 52 (MRI)
	The change from baseline to week 52 in cartilage thickness of the target knee tFTC (MRI)
	The change from baseline to week 28 and to week 52 in bone area of the medial femoral condyle surface of the target knee (MRI)
	The change from baseline to week 52 in JSW of the target knee (X-ray)
Clinical efficacy endpoints	The change from baseline to week 52 in target knee WOMAC total score and subscale scores of pain, function and stiffness^a^
	The change from baseline to week 52 in pain (as measured on a 100 ​mm VAS)
	The change from baseline to week 52 in PGA (as measured on a 100 ​mm VAS)
	The proportion of participants who achieve an OMERACT–OARSI response^b^ at week 52
	Use of analgesic medication at every visit up to week 52
Safety endpoints	The occurrence of adverse events and changes over time in vital signs, laboratory values, physical examinations, body weight and electrocardiogram parameters
Pharmacokinetic endpoints	Pharmacokinetics of S201086/GLPG1972

cMFTC, central medial femorotibial compartment; JSW, joint space width; MRI, magnetic resonance imaging; OMERACT–OARSI, Outcome Measures in Rheumatology–Osteoarthritis Research Society International; PGA, patient global assessment; tFTC, total femorotibial compartment; VAS, visual analogue scale; WOMAC, Western Ontario and McMaster Universities Osteoarthritis Index.

a WOMAC version LK3.1. Possible WOMAC subscale scores: pain, 0–20; stiffness, 0–8; physical function, 0–68. The WOMAC total score is the sum of the subscores (range, 0–96).

b An OMERACT–OARSI response is defined as an improvement in WOMAC pain or function of at least 50% relative to baseline and an absolute change of 20 points or more, or an improvement of at least 20% and an absolute change of 10 or more in at least two of WOMAC pain, function or PGA [35].

### 2.5 Methodological aspects

#### 2.5.1 Study site training

To ensure that imaging is standardized and comparable across multiple sites, a customized imaging manual will be developed and distributed to each study site by Bioclinica (Princeton, USA), describing MRI and X-ray image acquisition of the knee, techniques for accurate and reproducible patient positioning and instructions for routine quality control. Radiology technologists will also receive imaging training from Bioclinica. For a site to qualify to begin imaging knees of study participants, an MRI test knee scan must pass a quality review conducted by Bioclinica and Chondrometrics GmbH (Ainring, Germany).

#### 2.5.2 Radiographic assessment

Radiography for assessment of KL grading, OARSI JSN and medial disease will be performed at screening for all eligible knees. Radiographs will also be used to assess minimum and fixed-location medial joint space width (JSW) of the target knee at screening and week 52 (or at the premature withdrawal visit if the baseline measurement is carried out at least 9 months before). Fixed flexion, posteroanterior weight-bearing radiographs will be captured using the Bioclinica Synaflexer™ X-ray positioning frame [17] to standardize patient position and calibrate the image scale. Radiographs will be collected from study sites and read centrally using software based on active shape models developed by Bioclinica. Minimum JSW will be measured between the femoral condyle and the floor of the tibial plateau using the technique described by Buckland-Wright, et al. [18].

Assessment of radiographs will be carried out once both time points for a participant are available. The time points for an individual participant will be presented as a pair, but the visit order blinded, to ensure that the reader does not know which is the baseline time point, and which is the follow-up. Readings will be conducted by experienced musculoskeletal radiologists. Reader variability will be assessed by reviewing the JSW status scores at baseline and change in JSW scores at follow-up compared with baseline for 30 participants. The intra-class correlation coefficient will be used to establish the reader agreement level.

##### 2.5.2.1 Quality control procedures

Bioclinica will perform quality checks on all radiographs, including assessing images for proper patient positioning, correct radiographic projection with emphasis on the intermargin distance of the tibial plateau, complete anatomical coverage, proper exposure and the presence of artefacts.

#### 2.5.3 Magnetic resonance imaging acquisition and measurement

MRI will be used to assess the primary endpoint and secondary structural endpoints (Table 4). Imaging of the target knee will be carried out after confirmation of participant eligibility and before the first treatment, and at weeks 28 and 52 (or at any premature withdrawal visit if the previous measurement is carried out at least 2 months before).

The imaging protocol will include a sagittal 3D T1-weighted sequence with fat suppression, developed by Bioclinica. Scans will be sent to Chondrometrics for central quantitative analysis of cMFTC and total femorotibial compartment (tFTC) cartilage thickness using the Chondrometrics 3.0 software platform [19], and to Imorphics (Manchester, UK) for central analysis of the bone area [20]. Quantitative analysis of cartilage thickness and bone area will be performed blind to the time point and treatment.

##### 2.5.3.1 Quality control procedures

Before being sent for central analysis, MRI knee scans will be checked by Bioclinica for correct slice thickness, in-plane resolution, slice plane orientation and alignment, for any artefacts, and to ensure that the same knee coil and MRI scanner were used for baseline and follow-up scans. All scans received by Chondrometrics will be checked by an image quality control expert for: general quality (e.g. a sufficient signal and contrast-to-noise ratio); adherence to acquisition parameters (spatial resolution, flip angle, echo and repetition time); adequate fat saturation; full anatomical coverage; correct orientation; and absence of artefacts. Image adequacy will be confirmed by the reader assigned to perform the manual segmentation of the femorotibial cartilages. All segmentations will be checked by an expert reader and corrections of segmentations requested as needed.

##### 2.5.3.2 MRI test-retest procedures

Baseline and follow-up images of each knee will be processed by the same reader. Intra-reader variability will be assessed by test-retest MRI scans acquired at baseline and week 52 for one of the first three participants at each study site, with repositioning between acquisitions [21]. The reader will be blinded to the acquisition time point, to determine intra-reader test-retest error under the same conditions that apply to longitudinal MRIs (for which repositioning is a source of error). The root-mean square standard deviation and coefficient of variation will be determined to assess the intra-reader test-retest reliability. The availability of test-retest scans at baseline and follow-up will also allow determination of the smallest detectable change thresholds using the approach described previously [22], which will be used to classify knees as progressors or non-progressors.

#### 2.5.4 Clinical assessments

Patient-reported pain intensity in both knees will be assessed at screening using a 100 ​mm VAS. Target knee pain will then be assessed using the 100 ​mm VAS and Western Ontario and McMaster Universities Osteoarthritis Index (WOMAC) questionnaire at baseline and predefined intervals throughout the study. All five WOMAC questions on pain will be used to determine the pain subscore. Additionally, patient global assessment (PGA) disease activity will be evaluated using a 100 ​mm VAS. Participants' scores on the pain VAS, PGA VAS and WOMAC questionnaire will be recorded using an electronic patient-reported outcomes device. Participants’ use of analgesics for treatment of knee pain will be recorded at each visit on a specific page of the electronic case report form (eCRF); use of all other authorized concomitant treatments taken during the study will be documented on a separate page of the eCRF.

### 2.6 Data management, monitoring and auditing

Data will be collected in an eCRF and stored electronically in a secure database. Clinical sites will be monitored to ensure that participants’ rights and well-being are protected, the reported trial data are accurate, complete and verifiable, and that the study is conducted in a manner that complies with the trial protocol. Sites will be assessed before the start of the study and at regular intervals throughout. An audit may be carried out during the study or after study completion.

### 2.7 Safety evaluation

AEs, results of physical examinations, and changes over time in vital signs, laboratory values, body weight and electrocardiogram (ECG) parameters will be recorded throughout the study. AEs will be documented in the eCRF, and the seriousness, severity and relationship to the study treatment will be ascribed. ECG readings and laboratory parameters will be measured at predefined time points and analysed centrally by Banook Group (Nancy, France) and ICON (Farmingdale, NY, USA), respectively. A Data and Safety Monitoring Board will independently review the safety data at prespecified time points.

### 2.8 Pharmacokinetic analyses

Blood samples will be collected at prespecified time points (weeks 4, 12, 28, 40 and 52 or at early withdrawal) to determine S201086/GLPG1972 plasma concentrations for pharmacokinetic analyses. Plasma concentrations will be used to build a population pharmacokinetics model. A subgroup analysis will be carried out for Japanese vs non-Japanese patients.

### 2.9 Statistical analyses

#### 2.9.1 Sample size estimation

Based on the placebo results from the sprifermin proof of concept trial in patients with knee OA [23], we calculated that 852 participants (213 per treatment group) should provide a minimal power of 70% to detect that at least one dose of S201086/GLPG1972 is superior to placebo with a difference of 0.0825 ​mm (standard deviation ​= ​0.30 ​mm) in the primary endpoint at a two-sided significance level of 5%, using analysis of covariance (ANCOVA) adjusted for multiple testing by a Dunnett procedure. A treatment effect of 0.0825 ​mm corresponds to 75% of the expected cartilage loss in the placebo group (0.11 ​mm over 1 year [23]).

This approach, instead of using the same model as that for the primary analysis, was chosen given that the difference between calculations was very low (six patients per treatment arm), and to avoid making too many assumptions.

#### 2.9.2 Statistical analyses

Efficacy endpoints will be analysed for all enrolled and randomized participants (the modified randomized set). The estimated between-group mean difference in the change from baseline to week 52 in cartilage thickness of the cMFTC (primary endpoint) will be evaluated using a restricted maximum likelihood-based, mixed-effects model for repeated measures approach using all longitudinal observations at each post-baseline visit, preceded by a multiple imputation step for participants without post-baseline measurements (details of the multiple imputation procedure are given in Supplementary Material 1). The statistical model includes fixed and categorical factors of treatment, region (Asia and rest of the world), time and treatment-by-time interaction, as well as the continuous and fixed covariates of baseline (the MRI measurement for the primary efficacy endpoint before initiation of the study treatment) and time-by-baseline interaction. The time-by-baseline interaction will allow assessment of whether the evolution of the change from baseline of MRI measurements depends on the baseline value. An unstructured covariance structure will be used to model the within-patient errors.

Differences between treatment groups in the change from baseline to week 52 in WOMAC scores, VAS pain, VAS PGA disease activity and cartilage thickness of the tFTC will be assessed using the same method as for the primary endpoint. Differences between treatment groups in the proportion of structural progressors (participants with at least 8% cartilage loss in the cMFTC of the target knee at week 52) and the proportion of participants who achieve an Outcome Measures in Rheumatology (OMERACT)–OARSI response will be analysed using a logistic model, preceded by a multiple imputation step for missing data. Comparisons between treatment groups in changes from baseline in JSW and bone area will be assessed using ANCOVA. The number and proportions of participants who use analgesics/NSAIDs will be reported overall and by treatment group. To account for multiplicity, the Dunnett procedure will be used to control the family-wise error rate at 5%. Safety data will be analysed for all participants who receive at least one dose of the study drug, and reported using descriptive statistics. Analyses will be performed using SAS®version 9.4.

## 3 Discussion

The prevalence of knee OA is rapidly increasing [24]. Despite increased understanding of OA pathology, there is an unmet need for treatments that prevent disease progression. The novel anticatabolic ADAMTS-5 inhibitor S201086/GLPG1972 is in development as a potential DMOAD, and has yielded promising results in preclinical and early clinical studies [[11], [12], [13]]. To assess the efficacy and safety of this treatment in participants with OA, a large, multicentre trial is necessary. The international, randomized, double-blind, placebo-controlled ROCCELLA trial (NCT03595618) has been designed to determine whether S201086/GLPG1972 slows medial compartment cartilage loss in participants with knee OA.

The ROCCELLA selection criteria were chosen with the aim of enrolling participants who will experience substantial disease progression and cartilage loss when treated with placebo, and with consistent moderate to high baseline pain. Although previous DMOAD trials have specified KL grade 2 or 3 as the radiographic criterion for participant enrolment [9,23,25], OARSI JSN is also important for predicting rates of cartilage loss. For example, an observational study of 836 participants demonstrated that KL grade 2 knees with OARSI JSN grade 1 or 2 displayed significantly greater cartilage loss over 1 year than those without JSN [26]. Additionally, studies have shown that cartilage loss is more likely to occur in the medial compartment than in the lateral compartment, because the medial compartment has the highest weight-bearing burden [27]. An observational study of cartilage thickness change in patients with knee OA also demonstrated that unicompartmental JSN is associated with cartilage loss in the narrowed compartment [28]. Target knees in ROCCELLA will therefore be selected to have predominantly medial disease, KL grade 2 or 3 and medial tibiofemoral JSN-OARSI grade 1 or 2.

MRI will be used to assess cartilage thickness for determination of the primary endpoint (change from baseline to week 52 in cMFTC cartilage thickness). MRI is a well-established imaging tool for structural assessment in knee OA [29], and is increasingly used for evaluating the efficacy of DMOAD candidates in clinical trials [9,[30], [31], [32]]. The Foundation for the National Institutes of Health Biomarker Consortium Study demonstrated a potential association between reduced cMFTC cartilage thickness as measured by quantitative MRI and radiographic and pain progression in OA [33]; therefore, assessment of cartilage loss by MRI may be advantageous compared with other imaging modalities.

To ensure MRI-based assessments in ROCCELLA are reliable, imaging will be standardized across study centres. Indeed, the involvement of multiple sites is often a challenge for large international trials [34]. In the present trial, MRIs will be acquired at sites that use equipment from different vendors. Thus much effort will be invested in site training and imaging quality controls, including conducting test-retest MRI scans. These scans may provide insights into the reproducibility that can be expected from a large multicentre study.

The use of strict selection criteria should ensure only participants likely to experience sufficient rates of cartilage loss for detection of a treatment effect are enrolled. Together with the use of leading-edge medical imaging technology and stringent quality controls, this should help to ensure the results of ROCCELLA are highly reliable. The methodology used in this trial may help to inform future trials with anticatabolic DMOADs.

## Declaration of competing interest

OI, KB and MP are employees of the Institut de Recherches Internationales Servier (IRIS). HD is an employee of Galapagos NV. EvdA is an employee of and owns stock options in Galapagos NV. NS and TF are employees of Bioclinica, which received service fees for central reading of medical images during the conduct of the study. WW received grants and personal fees from Galapagos NV during the conduct of the study; and grants from Merck KGaA, Bioclinica, Medivir, TissueGene, the Foundation for the National Institutes of Health (FNIH), and personal fees from Chondrometrics GmbH outside the submitted work, and is a part-time employee and co-owner of Chondrometrics GmbH. PGC received personal fees from AbbVie, Bristol Myers Squibb, Eli Lilly, EMD Serono, Flexion Therapeutics, Galapagos NV, Genascence, Gilead, Novartis and Pfizer outside the submitted work. FE received grants and personal fees from Galapagos NV, and personal fees from Institut de Recherches Internationales Servier (IRIS) during the conduct of the study; and grants from Merck KGaA, Bioclinica, Medivir, TissueGene, the Foundation for the National Institutes of Health (FNIH), Samumed, Boston Imaging Core Lab and BMBF (Fed. Ministry of Education and Res.), and personal fees from Merck KGaA, Samumed, Roche, Medtronic, Novartis, Abbvie, ICM, Healthlink and Chondrometrics GmbH outside the submitted work, and is a part-time employee and shareholder of Chondrometrics GmbH.
