# Supplementary material for: The design of a randomized, placebo-controlled, dose-ranging trial to investigate the efficacy and safety of the ADAMTS-5 inhibitor S201086/GLPG1972 in knee osteoarthritis
Source: Osteoarthr Cartil Open. 2021 Aug 16;3(4):100209. doi: 10.1016/j.ocarto.2021.100209 (PMC9718074; doi:10.1016/j.ocarto.2021.100209)
Supplement: Multimedia component 1 [file mmc1.docx]

**Supplementary materials**

*Supplementary material 1. Multiple imputation procedures.*

For patients for whom there will be no post-baseline measurement of the primary endpoint (regardless of the timing of study discontinuation), because they cannot be considered through the MMRM, a multiple imputation procedure will be used to impute the missing evaluations, as a prior step, assuming that those patients would be in their randomized arm. A total of 100 imputed partially-complete data sets will be generated.

Multiple imputation inference involves three consecutives phases:

1. Imputation:

The missing pattern is assumed to be monotone, thus the regression method will be used to impute missing data. This will be performed under MAR hypothesis, by treatment group, using the region factor and taking into account the baseline score of the primary endpoint, based on patients for whom every post-baseline measurement is available. NB this imputation step might be preceded by a multiple imputation approach based on an MCMC method, in case of arbitrary missing patterns. A total of 100 imputed partially-complete data sets will be generated.

2. Analysis:

The same analysis as described above will be applied to each of the 100 imputed data sets.

3. Combination:

Statistical inferences will be generated by combining results from 100 analyses using Rubin’s formulae [1]. The multiple imputation estimator of the difference between each S201086/GLPG1972 dose and placebo is the average of the individual 100 estimators. The variance of the estimator is the combination of the between- and within-imputation variability [2]. NB for patients with only one missing post-baseline measurement and a monotone pattern, missing data will not be imputed. Those missing measurements will be handled through the MMRM.

**References**

[1] Rubin DB, Multiple Imputation for Nonresponse in Surveys, New York Wiley (1987).

[2] Kenward MG, Carpenter J, Multiple imputation: current perspectives, Stat. Methods Med. Res. 16 (2007) 199-218.
